# How does a protein’s structure spell the difference between health and disease? Our journey to understand glaucoma-associated myocilin

**DOI:** 10.1371/journal.pbio.3000237

**Published:** 2019-04-22

**Authors:** Raquel L. Lieberman

**Affiliations:** School of Chemistry and Biochemistry, Georgia Institute of Technology, Atlanta, Georgia, United States of America

## Abstract

Over 20 years ago, alterations to the protein myocilin were confirmed to be linked to a heritable form of the prevalent eye disease, glaucoma, and 10 years ago, my lab set out to develop a deeper understanding of myocilin in its normal and diseased state. We have made strides in understanding how genetic mutations in myocilin likely lead to disease, but unlocking myocilin’s biological function is still an elusive goalpost. Is normal myocilin unimportant in the human body? Are scientists using the wrong methods to study myocilin biology? Here, I discuss my scientific journey into understanding one small part of the fascinating organ that is the eye.

## Abstract 2

This Research Matters article by Raquel Lieberman provides an overview of her lab's basic research on protein myocilin, an eye protein implicated in glaucoma but whose normal function has remained shrouded in mystery for over 20 years.

## The amino acid sequence confers the 3D protein structure

A basic tenet in protein biochemistry is that the amino acid sequence of a protein dictates its 3D arrangement, and this correct “structure” is uniquely suited to perform its intended biological function in the context of the cell. For enzymes, which are a subset of proteins that catalyze chemical reactions, the surface of the structure often will display a region called an active site perfectly matched to the shape and chemical properties of the substrate. Many proteins are not enzymes, however. Nonenzymatic proteins provide rigidity to a cell or tissue by interacting with other proteins, or enable cellular communication that depend on time-sensitive modifications. The amino acid sequence and shape of a given protein dictate function for nonenzymatic proteins as for enzymes, but insights are often less obvious from the structure.

Any changes in the amino acid sequence of a protein can lead to structural changes to proteins or enzymes. Cells are set up to detect such changes and respond swiftly by attempting to degrade the abnormal protein. If cells succeed at triage, they survive, but the protein is unavailable to complete its function, and disease consequences are termed a loss-of-function. If cells fail to remove the aberrant protein, a common outcome is cellular toxicity and even cell death, and the resulting disease phenotype is a so-called toxic gain-of-function.

## Human diseases caused by aberrant protein structure

The list of protein conformational diseases arising from a toxic gain of function are numerous and include prevalent and challenging disorders such as Alzheimer, Parkinson, and prion disease (Mad Cow/Creutzfeldt Jakob). A common feature of these diseases is that they have an early-onset familial subtype that involves genetic alterations in a specific protein, as well as a later-onset sporadic form that affects significantly more individuals; both exhibit a similar endpoint patient phenotype. By focusing on how a protein leads to the heritable form of the respective disease, researchers typically have obtained clues into the molecular basis of the disorder. Still, identifying the precise molecular culprit in the cascade of events following the gain of function leading to the associated disease outcome has not been straightforward for researchers, and debates continue. Understanding the normal function of genetically associated proteins in such diseases has generally been marginalized due to the fascinating and complex roles these proteins play in their respective disorders.

## A new addition to the list of protein conformational disorders: Glaucoma

This backdrop sets the stage for a project I embarked upon when I started my independent research lab about a decade ago. I came across a collection of papers describing the effect of mutations on myocilin, which cause the inherited form of glaucoma, a prevalent and heterogeneous disease leading to irreversible blindness. Familial, inherited genetic mutations in myocilin, found in populations throughout the world, account for about 3% to 5% of the 70 million cases of glaucoma worldwide. The disease is autosomal dominant—it only takes one bad copy of the myocilin gene to cause glaucoma. In addition, although myocilin is found throughout the human body, it does not cause a systemic disease. Familial mutations in myocilin appear only to cause significant damage to one particular, microscopic, anatomical eye tissue, the trabecular meshwork (TM), found in the anterior eye near the lens and iris. In its normal state, myocilin is secreted at relatively high levels to the TM, and the TM is diseased in most forms of glaucoma. The anterior eye does not have blood vessels, so the role of the TM is to drain nourishing fluid produced by the ciliary body near the lens. When fluid drainage is slowed, eye pressure increases, and this is a causal risk factor for glaucoma.

Genetic mutations lead to the production of myocilin with amino acid substitutions, predominantly within one segment of myocilin called the olfactomedin (OLF) domain. The production of myocilin with a single incorrect amino acid is a scenario that cannot be tolerated by cells found within the TM. The event leading to the toxic gain of function is myocilin aggregation. TM cells attempt to remove this mutant aggregating protein but fail. Uncontrolled accumulating mutant protein, which is probably due to the specific type of aggregate, called amyloid, that is common to the aforementioned protein conformational diseases, leads to TM cell toxicity and eventually death. Without cells to maintain the TM tissue, ocular fluid flow is impeded and eye pressure cannot be adequately controlled. A major collaborative effort in my lab surrounds converting this knowledge into a new therapeutic strategy to treat glaucoma.

## What about the biological function of myocilin?

As with other protein conformational disorders, working out the role that mutant myocilin plays in glaucoma has had higher priority than identifying the biological function of myocilin in its normal, wild-type state. We have succeeded in piecing together the architecture of myocilin and the effects of amino acid substitutions at the molecular level, using molecular biophysics techniques such as X-ray crystallography and small angle X-ray scattering. Myocilin takes on a unique Y-shape, with two pairs of OLF domains clustered at the tips of the Y, and a coiled region at the stem, connected by a long linker. We have tested our chemical intuition regarding the effects of changing any particular amino acid, namely, the propensity of any given variant to aggregate and thus lead to glaucoma.

The Y molecular shape and surface charge of wild-type myocilin must be clues into what shapes might be complementary to productively interact with myocilin, but that’s where our explicit insight ends. Mice lacking myocilin do not exhibit a strong phenotype, and individuals with a truncation mutation that prevents myocilin from being made into a protein do not have identifiable systemic disease. Other OLF-domain–containing proteins provide some inspiration. For example, we might expect an elongated shape for an interacting partner based on parallels with an OLF family member found in nerves of the central nervous system. Or the long linker within myocilin might be cleaved, dividing the protein into parts, as seen for another OLF family member found in the peripheral nervous system. Overall, if the explicit function of myocilin, or its binding partners, could be readily identified, it would have been already: this paragraph summarizes the most recent of 20 years of research on myocilin biology at the molecular level.

## Our proposed strategy to crack the myocilin function “nut”

If researchers could identify stable binding partners for other OLF family members, researchers studying myocilin can too. Is myocilin unimportant? One interpretation of the apparent weak phenotype in humans and mice lacking myocilin is that myocilin is dispensable, but this is almost certainly not the end of the story. Cells do not waste precious energy and resources making macromolecules that are not needed, particularly in high quantities, and especially in cells that are long lived and don’t divide like TM cells.

We think that unlocking the mystery of myocilin’s binding partners and function in the TM could give us new clues enabling a better understanding of myocilin function in normal physiology, as well as the role of myocilin misfolding in normal eye aging, glaucoma, and even other scenarios in the body that may be overlooked. My lab’s approach to this problem is to start, in some sense, from scratch and revamp the research reagents scientists have to study myocilin. Our fundamental structural and biophysical insights have enabled us to pinpoint limitations in the suite of commercial reagents researchers have on hand for their studies. Our plan is to focus on developing new reagents that can uniquely identify different parts of the myocilin Y and that read out whether myocilin is in its normal state or one that could be disease linked. These new reagents, subsequently used by us and others in myriad basic and translational science experiments, will reveal significant new insights about the complex and fascinating TM eye tissue that is diseased in most forms of glaucoma. In the long run, we hope that our basic science, bottom-up approach will have an impact on our ability to understand and protect human sight.

## Abbreviations

OLF: olfactomedin
TM: trabecular meshwork

